# Deconvolution for multimode fiber imaging: modeling of spatially variant PSF

**DOI:** 10.1364/BOE.399983

**Published:** 2020-07-29

**Authors:** Raphaël Turcotte, Eusebiu Sutu, Carla C. Schmidt, Nigel J. Emptage, Martin J. Booth

**Affiliations:** 1 Department of Engineering Science, University of Oxford, Parks Road, Oxford OX1 3PJ, United Kingdom; 2 Department of Pharmacology, University of Oxford, Mansfield Road, Oxford OX1 3QT, United Kingdom

## Abstract

Focusing light through a step-index multimode optical fiber (MMF) using wavefront control enables minimally-invasive endoscopy of biological tissue. The point spread function (PSF) of such an imaging system is spatially variant, and this variation limits compensation for blurring using most deconvolution algorithms as they require a uniform PSF. However, modeling the spatially variant PSF into a series of spatially invariant PSFs re-opens the possibility of deconvolution. To achieve this we developed svmPSF: an open-source Java-based framework compatible with ImageJ. The approach takes a series of point response measurements across the field-of-view (FOV) and applies principal component analysis to the measurements' co-variance matrix to generate a PSF model. By combining the svmPSF output with a modified Richardson-Lucy deconvolution algorithm, we were able to deblur and regularize fluorescence images of beads and live neurons acquired with a MMF, and thus effectively increasing the FOV.

## 1. Introduction

Micro-endoscopes based on the insertion of a step-index MMF in living tissue have emerged as an alternative to systems utilizing graded index (GRIN) lenses [1–3]. This approach is particularly suitable for *in vivo* fluorescence imaging when minimal invasiveness is required, e.g. brain imaging. Indeed, the smaller diameter of MMFs compared to GRIN lenses decreases the displaced volume by 25-100 fold, thus substantially reducing tissue damage and physiological alteration. Key to this technology is the use of a spatial light modulator for modulating the wavefront such that the output field can be controlled, even as unpredictable distortions of the input field occur inside the MMF. Using wavefront control and taking advantage of the deterministic quality of the distortions for a given MMF segment, diffraction-limited foci can be formed in the specimen at the distal end of a MMF [4,5].

The ability of such systems to achieve sufficient spatial resolution for subcellular imaging is of particular interest in neuroscience where resolving subcellular features, such as dendritic spines, is essential for investigating biological processes [6,7]. However, the effective numerical aperture (NA) at different distal locations is determined by a number of parameters: wavelength, fiber geometry, and refractive indices (core, cladding and distal medium). While the specified NA can be utilized for focusing in a conical volume near the fiber tip, the effective NA decreases when moving axially away from the tip and radially from the center of the core [8]. This latter variation in NA causes the focus to spatially vary throughout a single FOV. In turn, the spatially varying point response limits our ability to perform linear filtering, including deconvolution, which could be used to deblur images, but also to regularize the point response.

Methods relying on modeling the spatially variant PSF have been shown to enable deconvolution using fast Fourier transform [9–16]. Such methods have been employed in a wide-range of biomedical applications: widefield fluorescence microscopy [10,11], structured illumination microscopy [10], localization microscopy [12], optical tomography [13], and near-infrared fluorescence imaging [14]. In several cases, the spatial variation was assumed to occur exclusively along the axial direction [15,16], enabling the use of two-dimensional deconvolution methods [10,11]. This approach is not adequate for MMF systems due to the presence of radial variations. Blind and semi-blind image restoration methods have also been proposed [17,18], but spatial variations in MMF systems can be readily measured without additional instrumentation, thus substantially simplifying the computational task. Machine learning methods also have the potential to model a spatially variant PSF and perform deconvolution in this context [18]. However, these approaches unnecessarily increase the computational complexity when a relatively simple analytic approach is also available, which is the case for MMF. In addition, the learning step of machine learning methods is likely to require substantially more data than an analytic solution.

In this work, we developed an open-source modeling framework, svmPSF, based on modal PSF modeling [19,20]. This method was selected because it is expected to be particularly well-suited for the smooth PSF variations encountered in MMF micro-endoscopy. In addition, because of the rotational symmetry of MMF systems, a limited number of modes are likely to be sufficient to represent most of the spatial variations, thus minimizing computing requirements. After describing the framework and characterizing its performance, we assessed the performance of a Richardson-Lucy deconvolution algorithm with total variation regularization based on modal PSF modeling in deblurring fluorescence images acquired with a MMF imaging system.

## 2. Methods

### 2.1 Experimental system

**Fig. 1.**
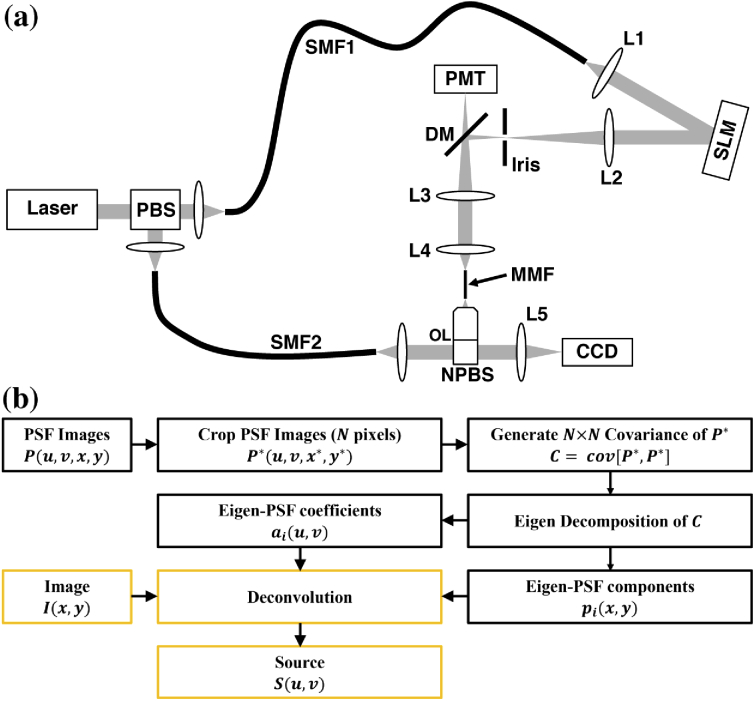
(a) Schematic of the experimental system. (b) Simplified flowchart for modeling a spatially-variant point response (black), including the additional implementation of deconvolution (yellow).

The MMF-based optical setup operated primarily as a one-photon fluorescence point-scanning microscope (Fig. 1(a)) [1,5]. The light from a continuous-wave laser (CrystaLaser, DL488-020-S, 488 nm) was delivered to a liquid-crystal spatial light modulator (SLM, Meadowlark Optics, HSPDM 512) using a single-mode optical fiber (SMF1, Thorlabs, P1-488PM-FC-2) and a collimating lens (L1, Edmund Optics, #47-636). The SLM shaped the wavefront of the first-order diffraction beam, which was aligned on-axis, and was conjugated (L2, Edmund Optics, #47-641) to an aperture to block other diffraction orders. The aperture was re-imaged (L3, Edmund Optics, #47-637 and L4, Thorlabs, C240TME-A) onto the proximal fiber facet. Light was thus coupled into the MMF and focused into a single point to excite fluorophores. A quarter-wave plate (Thorlabs, WPQ05M-488) located just before L4 made the polarization of the light entering the MMF circular. The fluorescence was collected by the MMF, directed to a photo-multiplier tube (Thorlabs, PMM02) using L3 and L4, and separated from the illumination by a dichroic mirror (DM). To form an image, a sequence of wavefronts was generated such that adjacent locations would be illuminated, effectively raster scanning the illumination focus.

**Fig. 2.**
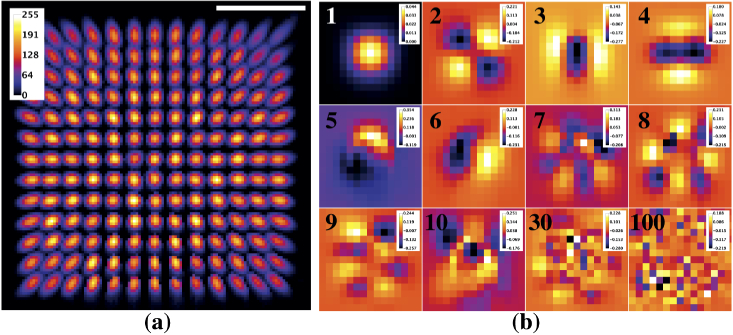
spatially variant point responses can be modeled continuously over the FOV. (a) Maximal intensity projection of experimentally measured foci through a MMF used to generate the eigen-PSF model (Scale bar: 15 µm). (b) Images of individual modes composing the eigen-PSF model for the data in (a) (mode number: 1-10, 30, and 100 modes; image width: 6.5 µm; color-bar units: [1]). A high-resolution version is available as Visualization 1.

The wavefronts were determined using the transmission matrix method, which characterizes the complex transformation between two planes. The input plane was defined as a grid of 69 × 69 points at the proximal facet of the MMF. The output plane was the targeted imaging plane located some distance away (35-75 µm) from the distal facet of the MMF. To determine the transmission matrix the output plane was re-imaged onto a CCD camera (Basler pilot, piA640-210gm) as a grid of 120 × 120 pixels by a microscope objective lens (OL, Olympus 20×, RMS20X, NA 0.4) and an achromatic doublet lens (L5, Thorlabs, AC254-125-A-ML). The polarization of the light from the MMF was converted to linear with a quarter-wave plate and merged with a reference signal using a 50:50 non-polarizing beam-splitter (NPBS). The reference signal consisted of light from the continuous-wave laser brought to the calibration assembly using a single-mode optical fiber (SMF2, Thorlabs, P1-405B-FC-5). A near-infrared version of this experimental system with a continuous-wave laser operating at 830 nm was used for the data shown in Fig. 1-2 [21].

### 2.2 PSF modeling algorithm

A PSF model can be generated using a zonal or modal representation [9,19,22,23]. The modal PSF model employed in svmPSF is based on the algorithm developed by Lauer [19] and is summarized in Fig. 1(b). When an imaging system has a spatially variant point response, the relation between the source S(u,v) and the image I(x,y) can no longer be expressed as a convolution but instead takes the form of:

(1) $$I(x,y)=\int_{-\infty }^{\infty }\int_{-\infty }^{\infty }S(u,v)\;P(u,v,x-u,y-v)\;du\;dv\;,$$

where the PSF P is dependent on both the shift (x−u and y−v) and the absolute position (u and v) in the source plane. The strategy proposed by Lauer was to separate the variables by expressing the PSF as a sum of orthogonal and spatially invariant PSFs obtained from the eigen-decomposition of focal spot measurements' co-variance matrix:

(2) $$P(u,v,x,y)=\sum_{i=1}^{N}a_{i}(u,v)\;p_{i}(x,y)\;,$$

where the $a_{i}(u,v)$ coefficients encode the spatial variability and N is the number of modes. For linear processing, the coefficients act as a weighting over the spatial domain of the source:

(3) $$I(x,y)=\sum_{i=1}^{N}\int_{-\infty }^{\infty }\int_{-\infty }^{\infty }S(u,v)\;a_{i}(u,v)\;p_{i}(x-u,y-v)\;du\;dv\;.$$

The two outputs of svmPSF are the eigen-PSFs $p_{i}$ and the coefficients $a_{i}$. The co-variance matrix was built by cropping an area of 15×15 pixels around each measured focus (see Section 2.3). The coefficients were calculated between the grid points using bicubic interpolation.

### 2.3 Focus recording

The input data required for PSF modeling, P(u,v,x,y), was generated by recording illumination focal spots sequentially on the CCD camera. No reference beam was present. The recording was accelerated by using the graphical processing unit for grabbing images recorded by the camera [5]. Equidistant grids of 13×13 or 21×21 points were recorded 3 times and then averaged together to ensure that any intensity variations were primarily due to the quality of the calibration, instead of noise. Performing a single measurement would likely have been sufficient as eigen-value decomposition will separate noise from true spatial variance, effectively denoising the PSF model. Images were composed of 120×120 pixels and the pixel size was 0.435 µm/pixel. A MATLAB script was used to numerically evaluate the $1/e^{2}$ width.

### 2.4 Sample preparation and imaging

Fluorescent beads (Invitrogen, F8823, yellow-green 505/515) having a diameter of 1 µm were imaged with a MMF having a numerical aperture (NA) of 0.66 and a core diameter of 44 µm. The custom-made MMF had a cladding of 6 µm and was encapsulated in an rigid tubing bringing the total diameter to 160 µm (Doris Lenses, Mono Fiberoptic Cannula, MFC_044/050150-0.66_5mm_ZF1.25_FTL). The rigidity tubing prevented any bending of the MMF. Without the external tubing, the MMF would be too flexible and easily deformed by air currents, thus changing the imaging area and altering the transmission matrix of the system. The calibration was performed at 35 µm from the distal end of the MMF and beads were imaged in air [24].

Organotypic hippocampal slices (350 µm) were prepared from male Wistar rats (P7-P8; Harlan UK) as previously described [21,25]. After dissection, slices were cultured on Millicell CM membranes and maintained in culture media at $37^{\circ }$C for 7-14 days prior to use. For imaging experiments, CA1 pyramidal neurons were loaded with Alexa Fluor 488 (2 mM) using whole-cell patch electrophysiology. During cell-filling, slices were superfused with oxygenated (95% $O_{2}$/5% ${\mathrm{CO}}_{2}$) artificial cerebrospinal fluid (ACSF; composition in mM: 145 NaCl, 2.5 KCl, 2 ${\mathrm{MgCl}}_{2}$, 3 ${\mathrm{CaCl}}_{2}$, 1.2 ${\mathrm{NaH}}_{2}$${\mathrm{PO}}_{4}$, 16 ${\mathrm{NaH}}_{2}$${\mathrm{CO}}_{3}$, 11 glucose). During imaging, slices were kept in physiological Tyrode’s solution (in mM: 120 NaCl, 2.5 KCl, 30 glucose, 2 ${\mathrm{CaCl}}_{2}$, 1 ${\mathrm{MgCl}}_{2}$, and 25 HEPES; pH = 7.2-7.4) at room temperature. Imaging was done using a MMF having a NA of 0.22 and a core diameter of 50 µm. The plane 50 µm away from the distal was calibrated with the fiber tip in Tyrode's solution [24].

### 2.5 Richardson-Lucy deconvolution

To deconvolve images acquired with an optical system having a spatially variant PSF, we implemented a modified version the Richardson-Lucy deconvolution algorithm as a stand-alone MATLAB script. Each iteration k of the algorithm evaluated the following equations:

(4) $$\begin{array}{r} TV_{k}=1/(1-\lambda_{TV}\cdot div\left[\frac{\nabla S_{k}}{|\nabla S_{k}|}\right])\;, \end{array}$$

(5) $$\begin{array}{r} I_{k}^{*}=\sum_{i=1}^{N}F\{p_{i}\}\cdot F\{a_{i}\cdot S_{k}\}\;, \end{array}$$

(6) $$\begin{array}{r} R_{k}=I/F^{-1}\{I_{k}^{*}\}\;, \end{array}$$

(7) $$\begin{array}{r} E_{k}^{*}=\sum_{i=1}^{N}F\{p_{i}^{\wedge }\}\cdot F\{a_{i}\cdot R_{k}\}\;, \end{array}$$

(8) $$\begin{array}{r} S_{k+1}=TV_{k}\cdot F^{-1}\{E_{k}^{*}\}\cdot S_{k}\;, \end{array}$$

where F and $F^{-1}$ are Fourier and inverse Fourier transforms, respectively, $R_{k}$ is the ratio of the image I over the estimated image $I_{k}$ ($I_{k}^{*}$ being the estimate frequency space image) at iteration k, and $E_{k}^{*}$ is the frequency space correction to apply to the current source estimated $S_{k}$ to get the next estimate $S_{k+1}$. A term for total variation regularization $TV_{k}$ with the $l_{1}$ norm was included to alleviate the effect of noise [26]. The value of $\lambda_{TV}$ was 0.002, except when processing neuron images ($\lambda_{TV}=0.02$). Of note, the Richardson-Lucy algorithm was modified to consider the PSF model when convolving the source estimate $S_{k}$ to get the image estimate $I_{k}$ Eq. (5) but also when computing the correction $E_{k}^{*}$ from $R_{k}$ Eq. (7) by correcting $S_{k}$ or $R_{k}$ with $a_{i}$ and then summing over N modes the convolution with eigen-PSFs. A Gaussian filter (2/3 pixel) was applied after deconvolution to minimize pixelation effects due to limited spatial sampling of the PSF during imaging. The FOV enhancement was calculated as the ratio of the circular area in which the peak intensity of the deconvolved point response was above a normalized value of 0.8 after deconvolution (500 iterations) with 30 modes over a single mode.

### 2.6 Access to the plugin and source code

All resources are hosted publicly on github. The PSF modeling resources comprise the Java source code, example data sets if point recordings, and a ready-to-use version of the svmPSF plugin for ImageJ/Fiji. A user manual is also included and is alternatively accessible via the group website aomicroscopy.org. The deconvolution resources consist of 3 MATLAB functions implementing the modified Richardson-Lucy algorithm with total variation regularization presented above. An example script is also included. As it can be seen from Eq. (5) and (7), the computational requirement for deconvolution and the speed of the process scale roughly linearly with the number of modes, and deconvolution with a shift-invariant PSF is computationally equivalent to using a single mode.

## 3. Results

### 3.1 PSF modeling

First, we show an example of focus recording and modeling through a MMF having a NA of 0.22 and a core diameter of 50 µm at a distance of 75 µm from the tip of the fiber. The recorded focus grid of 13×13 resulted in 169 images. These images are the primary input of svmPSF and are presented here as a maximum intensity projection (Fig. 2(a)). We observed the expected circular symmetry with a radial decrease in effective NA from the core center outward. Another key input is the size of the square region to crop around each measured focus. This parameter defines the size of the co-variance matrix and therefore the maximal number of modes available to generate the orthogonal basis. We used a crop size of 15×15 pixels in order to fully capture the elongated foci at the edge of the FOV, which gave a maximum of 225 modes (eigen-PSFs). The final input is related to foci registration. The expected foci locations were read from file. This is an important element because an erroneous registration would distort the model. As skewing was absent due to usage of the transmission matrix method, the focus peak location was used to determine the central position of each crop region.

svmPSF has three outputs. The first one is a file containing the metadata (pointers to focal spot data, input parameters, processing time, etc.). The second output is the eigen-PSFs, $p_{i}$, ordered according to the amplitude of their eigen-value. Figure 2(b) shows some eigen-PSFs for the MMF data from Fig. 2(a). The first mode corresponded to the average PSF; its coefficient $a_{1}$ was 1 and uniform across the image. Modes 2 to 10 showed spatial patterns having progressively higher spatial frequency and effectively modulating the average PSF through their amplitude that can be positive or negative (see color-bars in Fig. 2(b) and Visualization 1). Beyond the tenth mode it became challenging to discern any regular spatial pattern (e.g. mode 30) and above the hundredth modes the amplitude between adjacent pixels appeared to be uncorrelated. The third output is the coefficients $a_{i}$, which were determined at every pixel by bicubic interpolation. It would also have been possible to use a parametric approach to calculate these coefficients. This parametrization could even have decreased the number of measurements required for achieving equivalent modeling performance as our current approach by taking advantage of the azimuthal symmetry, i.e. the point response only varies radially, arising from the geometry of the fiber. Nevertheless, a parametric approach would have required 1) regressing the coefficients onto the parametric model, 2) determining accurately the location of the MMF center within the FOV, and 3) building a model of the spatial variance for a pre-selected subset of eigen-PSFs. Ultimately, this approach might have improved the accuracy of $a_{i}$, but at the cost of substantially limiting the generalizability of the svmPSF framework to a limited number of well described spatial variation patterns.

### 3.2 Reconstruction of experimental foci

**Fig. 3.**
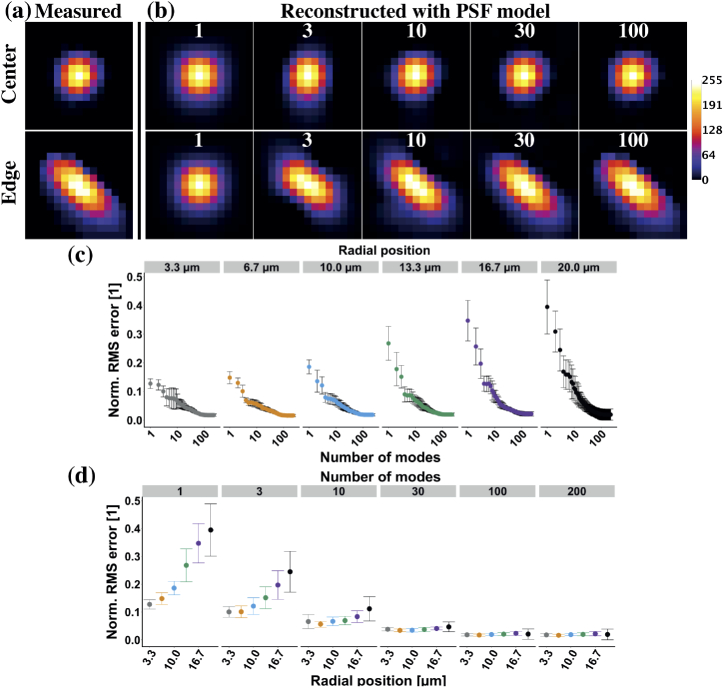
Experimental foci were reconstructed accurately using the eigen-PSF model. (a,b) Comparison between (a) the experimentally measured focus at the center (0 µm; top) and edge (20 µm; bottom) of the multimode fiber core and (b) the focus reconstructed from the eigen-PSF model with a varying number of modes (number of modes: 1-10, 30, and 100 modes; image width: 6.5 µm). (c,d) Difference, as the normalized RMS error, between the experimentally measured focus and the focus reconstructed from the eigen-PSF model (c) as a function of the number of modes used in the reconstruction for different distances from the center of the fiber and (d) as a function of the distance from the center of the fiber for different number of modes used in the reconstruction.

Next, we validated the model by comparing focal spots measured experimentally to the ones built using the outputs of svmPSF. The focal reconstructions were made using Eq. 2 in MATLAB and with a varying number of modes (from 1 to 225). Figure 3(a,b) shows an example in the center of the MMF and one at its edge. Using a single mode the reconstructions at both locations appeared different from the measured focal spots. The reconstructed central focal spot became indistinguishable from the experimental one with only ten modes and any additional mode did not contribute to any visible difference (Fig. 3(a,b) - top row). A larger number of modes (30) was required for the focal spot located at the edge as it was more different to the average PSF than the central one (Fig. 3(a,b) - bottom row). We quantified the difference between the experimental and reconstructed data by calculating the normalized root-mean-square (RMS) error between the two images as a function of the number of modes used in the reconstruction and the radial position from the center of the MMF (Fig. 3(c,d)). It is clear from the first panel (3.3 µm) of Fig. 3(c) that even near the center of the MMF the focal spots are not equivalent to the average PSF. Nevertheless, the first panel (1 mode) of Fig. 3(d) confirms that the difference between the average PSF and the measured focal spots increased with the radius. Figure 3(d) also reveals that a PSF model employing at least 30 modes would ensure spatial invariance (constant RMS error), whereas spatial variations are not fully accounted for in the PSF model when using 1, 3, and 10 modes as the RMS error increased.

### 3.3 Image deconvolution

Having demonstrated the accuracy of svmPSF in describing spatial variations, we then assessed the use of svmPSF outputs for image processing. Indeed, the motivation for a PSF model composed of spatially invariant PSFs was to enable linear filtering using Fourier methods. The convolution presented in Eq. (3) is an example of such an operation and appears twice in the Richardson-Lucy deconvolution algorithm. Therefore, we employed a modified version of this algorithm which took into account the spatial variance of the focus for image deconvolution (Section 2.5) and also included a regularization term to minimize the effect of noise.

**Fig. 4.**
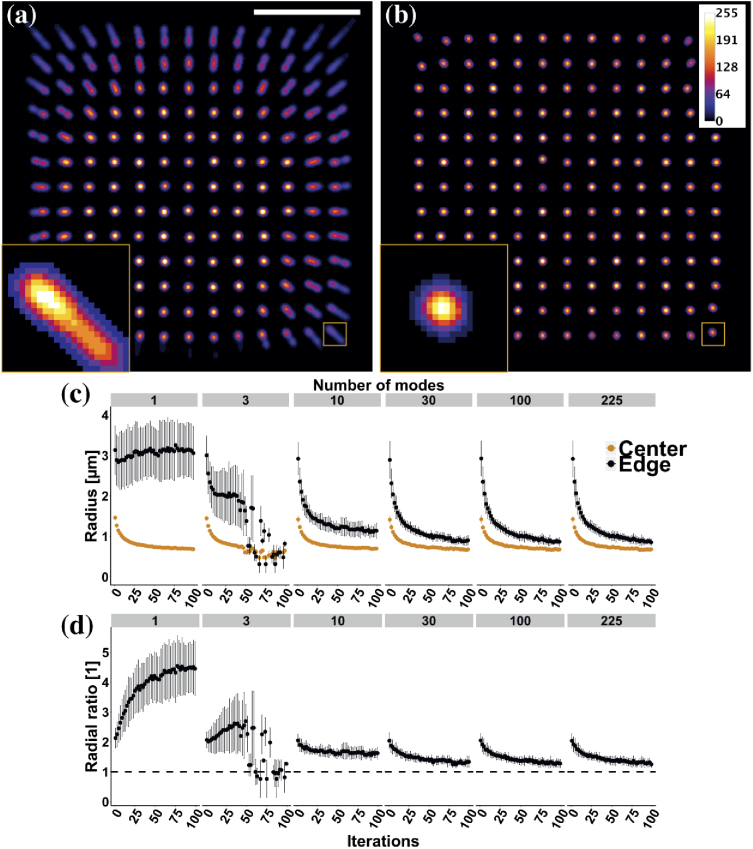
Spatial regularization of the point response is achieved through deconvolution using the svmPSF model. (a,b) Deconvolution of the image shown in Fig. 2(a) using a spatially variant version of the Richardson-Lucy algorithm after 500 iterations with (a) a single mode and (b) the first 30 modes (Scale bar: 15 µm; inset width: 3.3 µm). (c,d) Effect of the number of iterations and modes used in the deconvolution on (c) the $1/e^{2}$ radius of an object located at the center (3.3 µm) and edge (20 µm) of the MMF core and (d) the edge-to-center ratio of the radii in (c).

We started by deconvolving the image shown in Fig. 2(a) because the same data were used to generate the eigen-PSF model and the results thus provided information on the optimal performance of our approach. In addition, the uniform grid arrangement facilitated quantitative analysis. Figure 4(a,b) shows deconvolved images when using a single mode and 30 modes, respectively, after 500 iterations. In both cases, objects located in the center of FOV were similarly enhanced by deconvolution and preserved their circularity. When a single mode was used, deconvolution progressively increased the ellipticity of the objects as a function of the radial position (Fig. 4(a)), thus limiting the effective FOV to the central region (radius: 7 µm) in which symmetric foci were already present before deconvolution. When 30 modes were used, all objects acquired a symmetric shapes and were of a similar size, including objects located at the edge of the FOV (Fig. 4(b)). Beyond decreasing the apparent size of objects, deconvolution with the PSF model regularized the point response across the entire FOV; hence, the effective imaging area was increased by a factor of 3. Of note, the FOV improvement is dependent on the fiber and imaging geometry, in particular the distance from the MMF facet.

We then characterized the effect of the number of modes and iterations on the spatial profile of objects after deconvolution. The radius of objects at the center and edge of the FOV was quantified along the radial direction using the $1/e^{2}$ criterion (Fig. 4(c)). For objects at the center of the FOV, the change in radius was mostly independent of the number of modes. For objects at the edge of the FOV, no decrease in radius was achieved using a single mode. With fewer than 30 modes, some iterations yielded pixels with erroneously high intensity value, which caused the deconvolution to fail. This failure is visible here for 3 modes but would also be visible for 10 modes if more iterations were shown (Fig. 4(c,d)). No clear difference in radius as a function of the number of iterations were visible between 30 and 225 modes. The evaluation of the edge-to-center radial ratio as a function of the number of iterations showed an increase when fewer than 10 modes were used (Fig. 4(d)). In other words, the spatial non-uniformity was increased by deconvolution. For 10 modes, there is some improvement in uniformity as the ratio decreases, but the response is not regularized as the ratio does not approach 1. Again, no clear difference in this ratio as a function of the number of iterations was visible between 30 and 225 modes; the ratio was asymptotically approaching one with increased number of iterations. For this specific MMF and distal locations, the optimal number of modes for computation appears to be 30 as most of the variance would be accounted for while using only a fraction of the total number of modes in the PSF model.

**Fig. 5.**
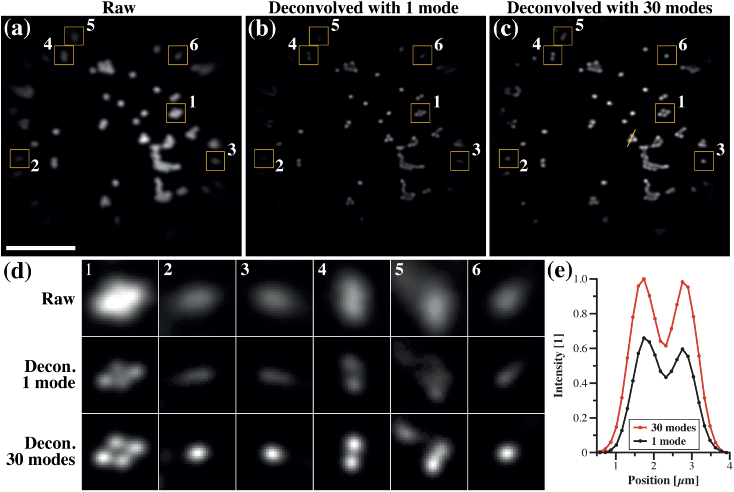
Spatially uniform deconvolution of fluorescent bead images having a spatially variant focus is achieved using the eigen-PSF model. (a) Image of 1-µm fluorescent beads acquired through a MMF (NA 0.66, 35 µm). (b,c) Deconvolved version of the image shown in (a) using a spatially variant version of the Richardson-Lucy algorithm after 500 iterations with (b) a single mode and (c) the first 30 modes (Scale bar: 15 µm). (d) Insets from (a-c) (width: 4.2 µm). All images were normalized to maximum intensity of the 30-modes inset. (e) Normalized intensity profile for the line shown in (c).

**Fig. 6.**
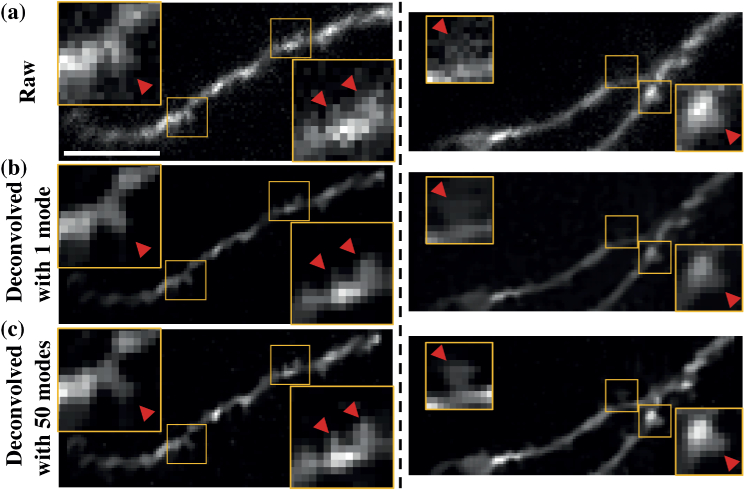
Deconvolution of neuronal images reveals fine subcellular details, such as spines (arrow). (a) Live neurons were imaged with a MMF (NA 0.22, 50 µm) and (b,c) deconvolved using the modified Richardson-Lucy algorithm (500 iterations and (b) 1 or (c) 50 modes). Two examples are shown (Scale bar: 15 µm). Insets were intensity normalized independently (left column inset width: 6 µm; right column inset width: 4.7 µm).

Finally, we tested the performance of the eigen-PSF model when employed to deconvolve fluorescence images acquired with a MMF. Focal recordings were acquired immediately following calibration and fluorescent beads of 1-µm in diameter were imaged with the MMF-based system (Fig. 5(a)). We then used the focal recordings to generate an eigen-PSF model that was specific to the fiber and system configuration. The images were deconvolved using the modified Richardson-Lucy algorithm with total variation regularization (500 iterations; 1 mode in Fig. 5(b) and 30 modes in in Fig. 5(c)). When 30 modes were used, images were successfully deblurred and the shape of structures improved through the process. For comparison, we provide a side-by-side montage of raw data and deconvolved data at different locations (Fig. 5(d)). The ellipticity of beads at the edge of the FOV was almost null with 30 modes in comparison to a single mode, which showed strong ellipticity. Individual beads within a cluster were also better separated, even in the center of the FOV, with 30 modes (Fig. 5(d,e)). In fact, the peak intensity of every bead was higher with 30 modes relative to 1 mode. Together, these results indicates that our svmPSF framework provided a PSF model enabling deconvolution and regularization of fluorescence images acquired on a system having a spatially variant point response. We therefore applied this approach to biological images of live neurons. The two examples shown illustrate how deconvolution enhanced small features such as spines that were often not visible in the non-deconvolved image (Fig. 6).

## 4. Discussion

The focus of this work was to create an open-source resource for PSF modeling when spatial variations are present (svmPSF) and to demonstrate its use to process imaging data acquired using a MMF. The PSF model provided by svmPSF was made to be compatible with linear filters based on Fourier methods. This point was illustrated through deconvolution, though deconvolution is only one form of linear filtering with Fourier methods. As several excellent tools already exist for deconvolution (e.g. [27]), we designed svmPSF to be stand-alone, i.e. not integrated into a deconvolution platform, for its optimal integration into existing workflows. Importantly, we observed that using an insufficient number of modes in the PSF model or assuming a uniform point response during deconvolution with the modified Richardson-Lucy algorithm exacerbated the spatial variance (Fig. 4). Of note, not all deconvolution algorithms are linear. For instance, Wiener filtering could not be enabled with svmPSF.

It is an inherent property that the PSF varies across the FOV of a MMF imaging system due to the focusing geometry. Experimental strategies have been devised to increase the uniformity of the focal spot across the FOV. One option consists in sacrificing spatial resolution by imaging away from the distal facet, beyond the region at which diffraction-limited resolution can be achieved. Of course, this approach will also significantly reduce signal collection and is suitable mainly when the goal is to visualize bright objects that are much larger then the diffraction limit such as cell bodies.

Alternatively, an optimal axial range closer to the distal facet can be selected, where diffraction limit is uniformly achieved in a region at the center of the FOV [1,3]. While this method has been useful in performing proof-of-principle studies for biomedical applications of MMF technologies, spatial variations will only become more severe as we seek to use MMFs with larger NA. Indeed, it is desirable to use MMFs with higher (>0.22) NA for multiple reasons. First, a high NA minimizes the effect of bending on the transmission matrix and therefore improves the robustness of the calibration [28]. Second, the ability to capture the morphology of subcellular objects, such as dendritic spines on neurons, is an important capability of MMF imaging systems, which will primarily be enabled through a substantial increase in NA. Third, advances in two-photon excited fluorescence microscopy through MMF will benefit from the use of high NA because of the nonlinear dependence between signal generation and NA [21,29]. In fact, even at equivalent NA the non-linearity of the two-photon process will result in increased spatial variations compared to one-photon fluorescence because the excitation volume is effectively the square of the illumination volume. In brief, it will likely be critical to perform regularization of the point response to achieve high-resolution, three-dimensional imaging over a large FOV using MMFs.

With respect to volumetric imaging, the proposed algorithm could be generalized in three dimensions for deconvolution. Alternatively, our two-dimensional model could be integrated with a strata model to achieve three-dimensional deconvolution [30]. In addition, we expect the algorithm to be applicable for extended depth-of-field imaging where axial symmetry is observed (e.g. Bessel beam) [31]. While we were able to demonstrate high quality deconvolution of images composed of objects well contained within the focal plane, and this was aided by the relative long depth-of-field of the system [1], the provided tools are limited to such source distributions for, clearly, three-dimensional deconvolution would be required for a three-dimensional source distribution.

## 5. Conclusion

In summary, we presented svmPSF, a Java framework for the modeling of spatially variant point responses in imaging systems. By using an approach based on eigen-value decomposition of the co-variance matrix, we were able to describe the spatial variation continuously across the FOV. In turn, this modeling enabled accurate reconstruction of focal spots and image deconvolution using a modified Richardson-Lucy algorithm with total variation regularization. In particular, the deconvolution rendered the point response uniform across the FOV, effectively extending the zone where diffraction-limited performance according to the specified NA could be achieved. While the performance of svmPSF was demonstrated in the context of MMF endoscopy, the framework is generic and can be utilized to model spatial variations in any two-dimensional optical system and is therefore expected to find usage beyond MMF applications.
