# Supplementary figures and images for: Deconvolution for multimode fiber imaging: modeling of spatially variant PSF
Source: Biomed Opt Express. 2020 Jul 29;11(8):4759–71. doi: 10.1364/BOE.399983 (PMC7449755; doi:10.1364/BOE.399983)

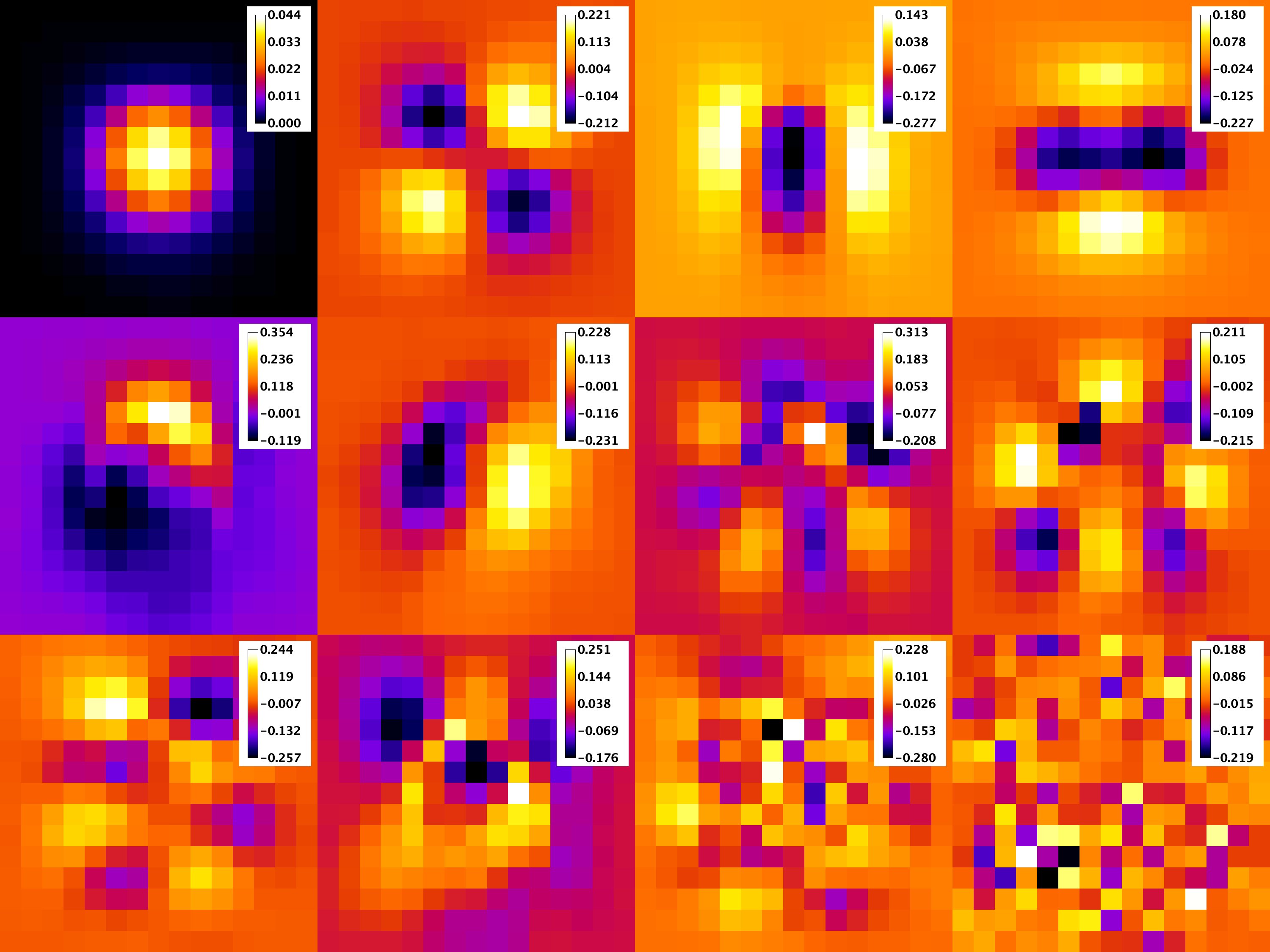

Supplement: Supplementary file 1 [file boe-11-8-4759-v001.jpg]
